# The Field of Science

**Published:** 1894-09-29

**Authors:** 


### The Field of Science.

The latest hygienic craze in Paris is the use of porous glass for windows. This is declared to possess all the advantages of the ordinary window framing, and, whilst light is as freely admitted as through the medium of common glass, the "porous" further admits air too, the minute holes with which this is intersected being too fine to permit of any draught, whilst they provide a healthy continuous ventilation through the apartment.

We are informed that a new hospital is to be established in Iceland. It was only the other day that we made reference to the Leprosy Commission which was shortly to be undertaken, and now, added to this, a well-equipped hospital, with resident doctor and nurses, is being discussed. The committee, a Danish one, at whose instigation the new scheme was formulated, are at work collecting the necessary funds to carry out this much-needed and praiseworthy undertaking in Iceland.

